# Effect of copper on nitrogen uptake, transportation, assimilation processes, and related gene expression in Chinese cabbage [*Brassica campestris* L. ssp. *Chinensis* (L.)] under various nitrate-to-ammonium ratios

**DOI:** 10.3389/fpls.2024.1427720

**Published:** 2024-09-25

**Authors:** Xin Wen, Peiran Xu, Yafang Tang, Hang Zhong, Pan Chen, Zhenhao Zhu, Xinya Zhang, Xiaohui Zhang, Aoran Du

**Affiliations:** ^1^ Zhejiang Ecological Civilization Academy, Anji, Zhejiang, China; ^2^ College of Environmental and Resource Sciences, Zhejiang University, Hangzhou, Zhejiang, China; ^3^ Hubei Key Laboratory of Quality Control of Characteristic Fruits and Vegetables, Hubei Engineering University, Xiaogan, Hubei, China; ^4^ School of Management, Minzu University of China, Beijing, China

**Keywords:** copper, nitrate/ammonium ratios, nitrogen metabolism, gene expression, Chinese cabbage

## Abstract

Improving vegetable yield and optimizing its quality through nutrient management have long been central to plant nutrition and horticultural science. Copper (Cu) is recognized as an essential trace element that promotes plant growth and development. However, the mechanisms by which Cu influences nitrogen (N) metabolism remain largely unknown, with limited studies exploring the interaction between Cu and varying nitrate-to-ammonium (nitrate/ammonium) ratios. In this study, Chinese cabbage was exposed to two Cu concentrations (0 and 0.02 mg L^-1^) in combination with three nitrate/ammonium ratios (10/90, 50/50, and 90/10) under hydroponic conditions. The results showed that Cu application increased plant biomass, nitrate reductase (NR) and glutamine synthetase (GS) enzyme activities, the expression of NR (*NIA*) and GS2 (*Gln2*) genes, and N content in both shoots and roots. Additionally, Cu treatment decreased nitrate and free amino acid contents, as well as the expression of nitrate transporters *NRT1.1* and *NRT2.1* in roots while increasing these four parameters in shoots. Additionally, these effects were significantly modulated by the nitrate/ammonium ratios. In conclusion, Cu may facilitate nitrate transportation, enhance nitrate reduction, promote ammonium assimilation, and influence the transformation of organic N compounds, highlighting its potential role in improving N metabolism in Chinese cabbage.

## 1 Introduction

Nitrogen (N) is a key driver of crop growth and development, playing a vital role in plant physiology and ecosystem functioning (Schulte-Uebbing et al., 2022; Tegeder and Masclaux-Daubresse, 2018; Moreau et al., 2019). In higher plants, nitrate and ammonium are the primary inorganic N sources. Once absorbed by roots, nitrate undergoes sequential reduction to ammonium via nitrate reductase (NR) and nitrite reductase (NiR). Ammonium is then assimilated into organic N through the glutamine synthetase-glutamate synthase (GS-GOGAT) cycle (Liu et al., 2022). The forms of available N significantly influence plant growth and nutrient absorption (Hernández Pérez et al., 2021). Most plants typically prefer nitrate as their N source, although they can absorb ammonium when nitrate is scarce (Naseri et al., 2022). However, high ammonium concentrations as the primary N source can induce toxicity, leading to fewer leaves, impaired root development, and reduced yield. Conversely, while nitrate promotes higher crop yields, it may cause nitrate accumulation in nitrate-preferring crops, especially leafy vegetables, potentially exceeding permissible limits (Luo et al., 2022). Thus, a balanced mixture of nitrate and ammonium is often recommended as an optimized N fertilization strategy. Compared to a single N source, an appropriate nitrate-to-ammonium (nitrate/ammonium) ratio significantly increases biomass in Chinese kale (Zhu et al., 2018), enhances soluble sugar, vitamin C, and soluble protein content in flowering Chinese cabbage (Song et al., 2017; Zhu et al., 2021), improves morphophysiological traits and phytochemical compounds in Moldavian balm (Naseri et al., 2022), alleviates heavy metal toxicity in grasses (de Souza Junior et al., 2018), and enhances cold stress resistance in tomato seedlings (Liu et al., 2017).

Copper (Cu) is an essential trace element for higher plants. It reportedly promotes carbohydrate metabolism (Ameh and Sayes, 2019) and is a key component of plastocyanin, which facilitates electron transfer in chloroplasts and mitochondria (Aguirre and Pilon, 2016). Cu also contributes to protein synthesis and transport (Ghazaryan et al., 2019), catalyzes redox reactions within plant cells (Da et al., 2019), and functions as a component or cofactor for numerous important enzymes (Zunaira et al., 2020). These physiological functions are closely linked to plant N metabolism, particularly in nitrate reduction. Cu is involved in the biogenesis of the molybdenum cofactor (Moco), a crucial component of NR, which maintains the enzyme’s spatial stability and functional efficacy (Kaur et al., 2023). Additionally, cytochrome c (Cyt c) oxidase, a Cu transport protein, participates in the electron transfer process where NR utilizes electrons from NAD(P)H to reduce acceptors like Cyt c (Chamizo-Ampudia et al., 2017). Carbohydrates produced during photosynthesis can induce the expression of NR-encoding genes (Xiong et al., 2006). Furthermore, plastocyanin, a Cu-containing protein, is integral to photosynthetic electron transfer (Zunaira et al., 2020). Numerous studies demonstrated that applying appropriate Cu concentrations regulates key N assimilation enzymes (Hristozkova et al., 2007), enhances symbiotic N fixation, and elevates nitrogenous metabolites like proteins and amino acids (Mazen, 2004; Xiong et al., 2006), thus increasing plant N content and accumulation (Seliga, 1995; Phan et al., 2009; Alhasany et al., 2019). However, few studies have explicitly determined which N form or nutritional ratio underlies these effects. The interaction between Cu and nitrate/ammonium ratio in higher plant growth has received limited attention, with available studies focusing on Cu pollution and toxicity (de Souza Junior et al., 2018). Consequently, research is lacking on the efficacy of combining optimal Cu concentrations with nitrate/ammonium nutritional ratios on higher plant growth and N metabolism.

Existing studies on the influence of Cu on plant N metabolism have primarily examined N component levels, metabolic products, and related enzyme activities. However, as plant growth, development, and nutritional responses are mediated through differential gene expression, exploring the nutritional functions of Cu in processes such as nitrate transport and reduction, and ammonium assimilation, from a gene expression perspective is essential. *NRT1.1* and *NRT2.1*, members of the low-affinity and high-affinity nitrate transporter families, respectively (Fan et al., 2017), function in nitrate transport at external nitrate concentration of > 0.5 mM and < 0.5 mM (Tsay et al., 2007; Miller et al., 2009). In *Arabidopsis* roots, *AtNRT1.1* and *AtNRT2.1* expression levels are strongly induced by nitrate (Okamoto et al., 2003). *NIA*, a key gene encoding the rate-limiting enzyme NR in nitrate reduction, is induced by carbohydrates, light, and nitrate, but inhibited by metabolic products like glutamine and other amino acids (Chamizo-Ampudia et al., 2017; Sun et al., 2018). The *Gln1* and *Gln2* gene families encode two GS isoforms with distinct subcellular localizations: cytosolic GS1 (three to five genes, species-dependent, e.g., *AtGLN1.1-1.5* in *Arabidopsis*, *OsGS1;1-1;3* in rice, *ZmGLN1.1-1.5* in maize, and *TaGS1.1-1.3* in wheat) and plastidial GS2 (a single gene, e.g., *AtGLN2* in Arabidopsis, *OsGS2* in rice, *ZmGLN2* in maize, and *TaGS2* in wheat) (Konishi et al., 2018; Wei et al., 2020; Liu et al., 2022). While extensive research has examined the induction of these genes by different N forms in various plants, such as *Arabidopsis* (Hachiya et al., 2021), maize (Prinsi and Espen, 2015), wheat (Wei et al., 2021), and citrange (Sun et al., 2018), studies on their responses to Cu or in Chinese cabbage are limited. Moreover, the interaction between Cu and nitrate/ammonium ratios and its impact on the expression of these genes remains unclear.

Chinese cabbage, a leafy vegetable native to China, is a member of the *Brassica* genus within the *Brassicaceae* family. Known for its nutritional value, it is rich in vitamins, minerals, amino acids, and dietary fiber. This nitrate-loving crop can yield significantly with pure or high nitrate fertilization, but this can lead to excessive nitrate accumulation in the edible parts (Mantovani et al., 2018). Adjusting the nitrate/ammonium ratio in fertilization measures can significantly decrease nitrate content in Chinese cabbage (Zhu et al., 2015). Brassicaceous crops, including Chinese cabbage, have a high demand for Cu, especially during the reproductive growth stage when they become sensitive to low copper stress. Cu deficiency significantly impacts N and carbohydrate metabolism in vegetables (Ameh and Sayes, 2019). This results in changes in the regulation of various genes controlling different process functions (Thomas et al., 2016), ultimately affecting vegetable growth, development, and overall quality. However, current research on Cu transport in Chinese cabbage is indeed limited, with the majority of studies focusing on the phytotoxic effects of excessive Cu levels rather than exploring the impact of appropriate Cu concentrations. Previous studies have demonstrated that a Cu concentration of 0.02 mg L^−1^ can enhance the biomass of Chinese cabbage, while concentrations reaching 0.27 mg L^−1^ inhibit growth (Aghajanzadeh et al., 2020; Schmitt et al., 2020), but these studies have primarily addressed growth metrics and have not investigated the effects of Cu-N interactions. Addressing these gaps is critical, especially in understanding how N forms and Cu application influence the cultivation of Chinese cabbage. Key areas for investigation include inorganic N content levels, enzyme activity, primary N assimilation products, N accumulation, key transporters, and gene expression. Expanding this research will enhance our knowledge of Cu’s role in enhancing nutritional functions and ensuring vegetable nitrate food safety. Thus, our study aimed to: (1) assess the effects of Cu on plant growth and nutrient N metabolism in Chinese cabbage under varying nitrate/ammonium ratios in the nutrient solution; and (2) determine Cu’s impact on the gene expression of nitrate transporters, NR, and GS in Chinese cabbage. Therefore, these findings offer insights into the mechanisms by which Cu enhanced plant N metabolism, suggesting strategies for optimizing nutrient management to improve vegetable yield and quality.

## 2 Materials and methods

### 2.1 Plant materials and growth conditions

Chinese cabbage [*B. campestris* L. ssp. *Chinensis* (L.). cv. *Shanghai green*] was sourced from China for germination. The seeds were sterilized with 0.5% NaClO solution and germinated in deionized water at 25 °C for five days. Following germination, the seedlings were transplanted into rectangular hydroponic plots and supplied with half-strength modified Hoagland and Arnon nutrient solution (Hoagland, 1950) for two weeks. The nutrient solution comprised 2 mM Ca(NO_3_)_2_·4H_2_O, 3 mM KNO_3_, 0.5 mM NH_4_H_2_PO_4_, 1 mM MgSO_4_·7H_2_O, 0.05 mM EDTA-Fe, 23.1 µM H_3_BO_3_, 4.55 µM MnCl_2_·4H_2_O, 0.4 µM ZnSO_4_·7H_2_O, 0.01 µM (NH_4_)_6_Mo_7_O_24_·4H_2_O, and 0.15 µM CuSO_4_·5H_2_O.

To assess the effects of Cu and nitrate/ammonium ratios on the growth and N assimilation in Chinese cabbage, a completely hydroponic trial was conducted using a 3×2 factorial treatment design. The Cu treatments included two concentrations: Cu 0 (0 mg L^−1^) and Cu 0.02 (0.02 mg L^−1^) representing Cu deficiency and Cu application, respectively. The nitrate/ammonium ratio treatments comprised three ratios: 10/90, 50/50, and 90/10. The macro-elements were based on Hoagland’s nutrient solution, containing 15 mM N, 1 mM phosphorus (P), 6 mM potassium (K), 5 mM calcium (Ca), and 2 mM magnesium (Mg) (**Table 1**). The trace elements were diluted to 1/1000^th^ of Arnon’s nutrient solution. For the Cu 0 group, CuSO_4_·5H_2_O was omitted from the nutrient solution, while for the Cu 0.02 group, 0.02 mg L^−1^ Cu was provided by adding CuSO_4_·5H_2_O at 0.08 mg L^−1^. Additionally, 7 μM dicyandiamide (DCD) was included to inhibit ammonium nitrification.

**Table 1:** The composition and formulation of N, P, K, Ca, and Mg in the nutrient solution under different nitrate/ammonium ratios.

Nutrient source	Nitrate/ammonium ratios in the nutrient solutions		
	10/90	50/50	90/10
Ca(NO_3_)_2_·4H_2_O	0.75	3.75	5
KNO_3_	/	/	1.75
KCl	5	5	3.25
KH_2_PO_4_	1	1	1
CaCl_2_	4.25	1.25	/
(NH_4_)_2_SO_4_	6.75	3.75	0.75
MgSO_4_·7H_2_O	2	2	2

When the second leaves of Chinese cabbage had fully expanded, the plants were transferred to plots containing 10 L of nutrient solution with varying Cu and nitrate/ammonium ratios, as previously described. The plants were randomly arranged in a greenhouse under controlled conditions: 60–80% relative humidity, 25°C during the day, 15°C at night, and 16 h of daily lighting at 500 mmol m^−2^ s^−1^. Each plot held 20 plants, with each treatment replicated thrice. All reagents used were of analytical grade (AR), and deionized water was utilized to prepare solutions, which were refreshed every seven days. The Chinese cabbage growth was continuously monitored and recorded throughout the incubation period.

After 45 days of growth, the plants were harvested and separated into shoots and roots. The tissues were washed with deionized water, thoroughly mixed, and then divided into two portions. One portion was rapidly frozen in liquid nitrogen and stored at −80°C for subsequent physiological analysis and RNA extraction. The other portion was weighed to determine fresh weight (FW), then subjected to heat treatment at 105°C for 30 min, followed by drying at 65°C to a constant weight to determine dry matter (DM). The dried samples were finely ground and sieved through a 0.85 mm mesh.

### 2.2 Total N, nitrate, and ammonium contents

To determine the total N content, 0.10 g of dried biomass was subjected to digestion using H_2_SO_4_-H_2_O_2_, as previously described (Wen et al., 2018, 2019). The N concentration in the diluted and filtered digested solutions was then measured using a Continuous-Flow Analyzer (SEAL Analytical, Germany). N accumulation was calculated as the N content in the dry plant biomass: N accumulation (mg) = N content × DM.

Nitrate content was determined using the previously reported method (Li, 2000). Briefly, 2.00 g of fresh samples were ground in 10 mL of ice-cold deionized water and subjected to a 30 min boiling water bath treatment. After cooling, 0.1 mL of the filtrate was aspirated and mixed with 0.4 mL of 5% salicylic-sulfuric acid solution. Following a 20 min incubation, 9.5 mL of 8% NaOH was added, and the absorbance was measured at 410 nm once the solution had cooled to room temperature.

Plant ammonium content was assessed using Nessler’s reagent colorimetric method at 480 nm, as described previously (Tang et al., 2013).

### 2.3 Analysis of N-assimilation enzyme activities and metabolite content

Two types of N-assimilation enzyme activities were analyzed using frozen samples (Wang, 2006) with minor modifications. NR (EC 1.7.1.1) activity was assessed as follows: Tissues (0.50 g) were ground in ice in 4 mL of extraction buffer comprising 0.1211 g KNO_3_, 0.0372 g EDTA, and 100 mL of 0.025 M phosphate buffer (pH 8.7). The homogenate was centrifuged at 4000 rpm for 15 min at 4°C, yielding the final supernatant. The reaction mixture included 1.2 mL of 0.1 M KNO_3_ phosphate buffer, 0.4 mL of NADH, and 0.4 mL of enzyme extract. The mixture was incubated in a 25°C water bath for 30 min, followed by adding 1 mL of sulfanilamide to terminate the enzyme reaction and 1 mL of naphthalene vinyl amine dihydrochloride to develop color. Absorbance was measured at 540 nm after 15 min. The GS (EC 6.3.1.2) activity was assessed as follows: Tissues (0.50 g) were ground in ice with 3 mL of 50 mM Tris-HCl buffer (containing 2 mM MgCl_2_, 2 mM DTT, and 0.4 M sucrose, pH 8.0). The homogenate was centrifuged at 1300 rpm for 10 min at 4°C, yielding the final supernatant. Following centrifugation, 1.6 mL of hydroxylamine hydrochloride and 0.7 mL of ATP solution were added to the supernatant, mixed, and incubated at 37°C for 30 min. Subsequently, 1 mL of a color reagent (containing 0.2 M TCA, 0.37 M FeCl_3_, and 0.6 M HCl) was added, and the absorbance was measured at 540 nm.

The soluble protein content was measured according to the method of Lu et al. (2014). Frozen tissue samples (0.20 g) were ground on ice with 2 mL of deionized water, and the homogenate was centrifuged at 4000 rpm for 20 min. From the supernatant, 0.1 mL was mixed with 5 mL of Coomassie Brilliant Blue G-250 Protein. The mixture was thoroughly blended, and absorbance was recorded at 595 nm.

Total free amino acid content was assessed using the ninhydrin colorimetric method (Li, 2000). Frozen tissue samples (0.50 g) were extracted with 10% acetic acid on ice, and the supernatant was reacted with deionized water and ninhydrin solution. The mixture was heated in a boiling water bath for 15 min, and then rapidly cooled with continuous agitation. After the bluish-violet color had developed, ethanol was added, and the solution was thoroughly mixed, with the absorbance being measured at 570 nm.

### 2.4 RNA extraction and qRT-PCR

Quantitative real-time reverse transcription PCR (qRT-PCR) was used to quantify the expression levels of the *NRT1.1*, *NRT2.1*, *NIA*, *Gln1*, and *Gln2* genes (Tang et al., 2013; Deng et al., 2024). Total RNA was extracted from 0.1 g of frozen shoots and roots using Trizol reagent (Invitrogen, USA). RNA concentration and purity were measured with a Nanodrop 2000 (Thermo, USA), and quality was assessed via 1% agarose gel electrophoresis. The RNA extracts were reverse-transcribed using M-MLV Reverse Transcriptase (Promega, USA) following the manufacturer’s instructions. The synthesized cDNA (50 ng) was diluted to 20 μL for qRT-PCR. The reaction was performed in technical triplicates using the SYBR Green PCR Master Mix Kit (TOYOBO, Japan) on a CFX96 Fast Real-Time PCR System (Bio-Rad, USA), with the *Actin* gene as an internal control. Primers used for qRT-PCR are listed in **Table 2**, with additional details provided in **Supplementary Tables S1**−**S4** and **Supplementary Figures S1**-**S6**.

**Table 2:** The primers used for qRT-PCR.

Target gene	Gene index No./Name	Primer type	Sequence (5’ to 3’)	References
*NRT1.1*	AJ278966	Forward	CTATATCGGTGGCCTCCTCCTA	(Zhao et al., 2010)
		Reverse	AGCTTTTTGCATAAGGGAAT	
*NRT2.1*	AJ293028	Forward	GGAGCACAAGCCGCTTGT	(Zhao et al., 2010)
		Reverse	AAGGGCTCGCCGAGAAAC	
*NIA*	NM_106425.2	Forward	GTTCGAGCCTGGGACGAGT	(Tabata et al., 2000)
	NM_103364.2	Reverse	GACGTTCCTTTGCCATCC	
*Gln1*	NM_123119	Forward	TCTTTAGCCACCCTGATGTTG	(Tabata et al., 2000)
		Reverse	TCTCCGTTGATGCCACTAATG	
*Gln2*	NM_122954	Forward	TGGAGCTGAAAAGTCTTGGG	(Tabata et al., 2000)
		Reverse	GTTCCAGTCACCCTCAATCG	
*Actin*	18S rRNA	Forward	AAACGCCTACCACATCCA	(Zhao et al., 2010)
		Reverse	CACCAGACTTGCCCTCCA	

### 2.5 Statistical analysis

Data analysis was conducted using SPSS 25.0 software. Results are presented as means ± standard errors in triplicate (n = 3). A two-way analysis of variance (ANOVA) was used to assess the main effects of nitrate/ammonium ratio and Cu levels, as well as their interaction effects. For all parameters, a *post-hoc* multiple comparisons were performed using Duncan’s test at a significance level of *p* < 0.05. Data visualization was carried out using the Origin 2021 software.

## 3 Results

### 3.1 Plant growth, nitrate, and ammonium contents

Cu application increased the biomass of Chinese cabbage across all nitrate/ammonium ratios, enhancing both FW and DM in shoots and roots (**Table 3**, **Supplementary Figure S7**). This effect was particularly significant at the 90/10 ratio (*p* < 0.05). The nitrate/ammonium ratio had a highly significant effect on root biomass, with both FW and DM consistently increasing as the nitrate proportion rose. In contrast, shoot FW and DM were significantly lower at the 50/50 ratio compared to the other ratios. Additionally, a significant interaction between Cu application and nitrate/ammonium ratio was observed, affecting shoot FW (*p* = 0.002) and DM (*p* < 0.001).

**Table 3:** Effects of Cu on the biomass of shoot and root of Chinese cabbage under different nitrate/ammonium ratios.

Nitrate/ammonium ratios	Cu addition (mg L^−1^)	Shoot (g plant^−1^)		Root (g plant^−1^)	
		FW	DM	FW	DM
10/90	0	9.76 ± 0.33 b	0.82 ± 0.01 b	2.71 ± 0.09 e	0.04 ± 0.00 d
	0.02	10.45 ± 1.31 b	0.88 ± 0.02 b	3.41 ± 0.12 d	0.06 ± 0.00 bc
50/50	0	7.53 ± 0.86 b	0.55 ± 0.00 c	3.72 ± 0.14 d	0.05 ± 0.00 cd
	0.02	8.45 ± 0.94 b	0.71 ± 0.01 bc	4.42 ± 0.20 c	0.07 ± 0.00 b
90/10	0	12.00 ± 0.70 b	0.93 ± 0.01 b	5.70 ± 0.17 b	0.07 ± 0.00 b
	0.02	22.06 ± 1.84 a	1.48 ± 0.02 a	7.14 ± 0.39 a	0.09 ± 0.00 a
*p*-value	Cu	0.003**	<0.001**	<0.001**	<0.001**
	N ratios	<0.001**	<0.001**	<0.001**	<0.001**
	Cu × N ratios	0.002**	<0.001**	0.171	0.531

Cu represents the main effect of Cu level, N ratios represent the main effect of nitrate/ammonium ratio, and Cu × N ratios represent the interaction effect of two factors. ** represents *p* < 0.01. Lowercase letters denote comparisons across each column; different letters indicate significant differences (*p* < 0.05).

**Figure 1:**
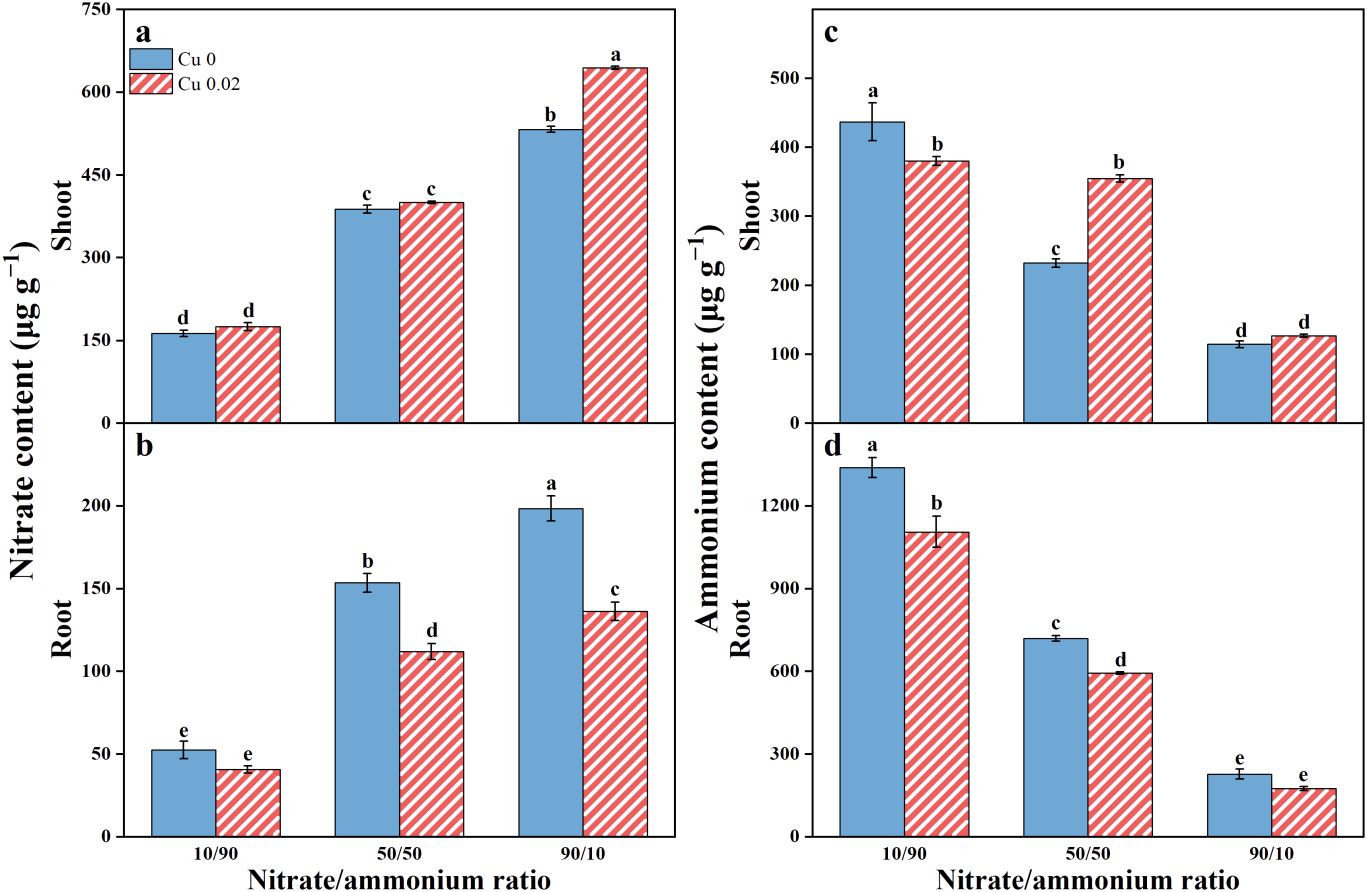
The effects of Cu on nitrate **(A, B)** and ammonium **(C, D)** contents in Chinese cabbage shoots and roots under three nitrate/ammonium ratios. Significance levels are denoted by distinct lowercase letters at *p* < 0.05.

As the nitrate proportion in the nutrient solution increased, nitrate content in Chinese cabbage also significantly increased. However, shoot and root responses to Cu varied (**Figures 1A, B**). Under the 90/10 ratio, Cu application significantly increased shoot nitrate by 20.92% (*p* < 0.05) but reduced root nitrate by 27.06% and 31.33% at the 50/50 and 90/10 ratios, respectively (*p* < 0.05).

In contrast, ammonium content in Chinese cabbage decreased progressively with increasing nitrate proportion (**Figures 1C, D**). Cu application significantly reduced shoot ammonium under the 10/90 ratio but increased it under the 50/50 ratio. Root ammonium content decreased across all nitrate/ammonium ratios, with significant reductions of 17.39% and 17.57% under the 10/90 and 50/50 ratios, respectively (*p* < 0.05).

Additionally, the nitrate content in the shoot of Chinese cabbage was significantly higher than in the root, while the ammonium content showed the opposite trend.

### 3.2 Gene expression of nitrate transporters *NRT1.1* and *NRT2.1*

**Figure 2:**
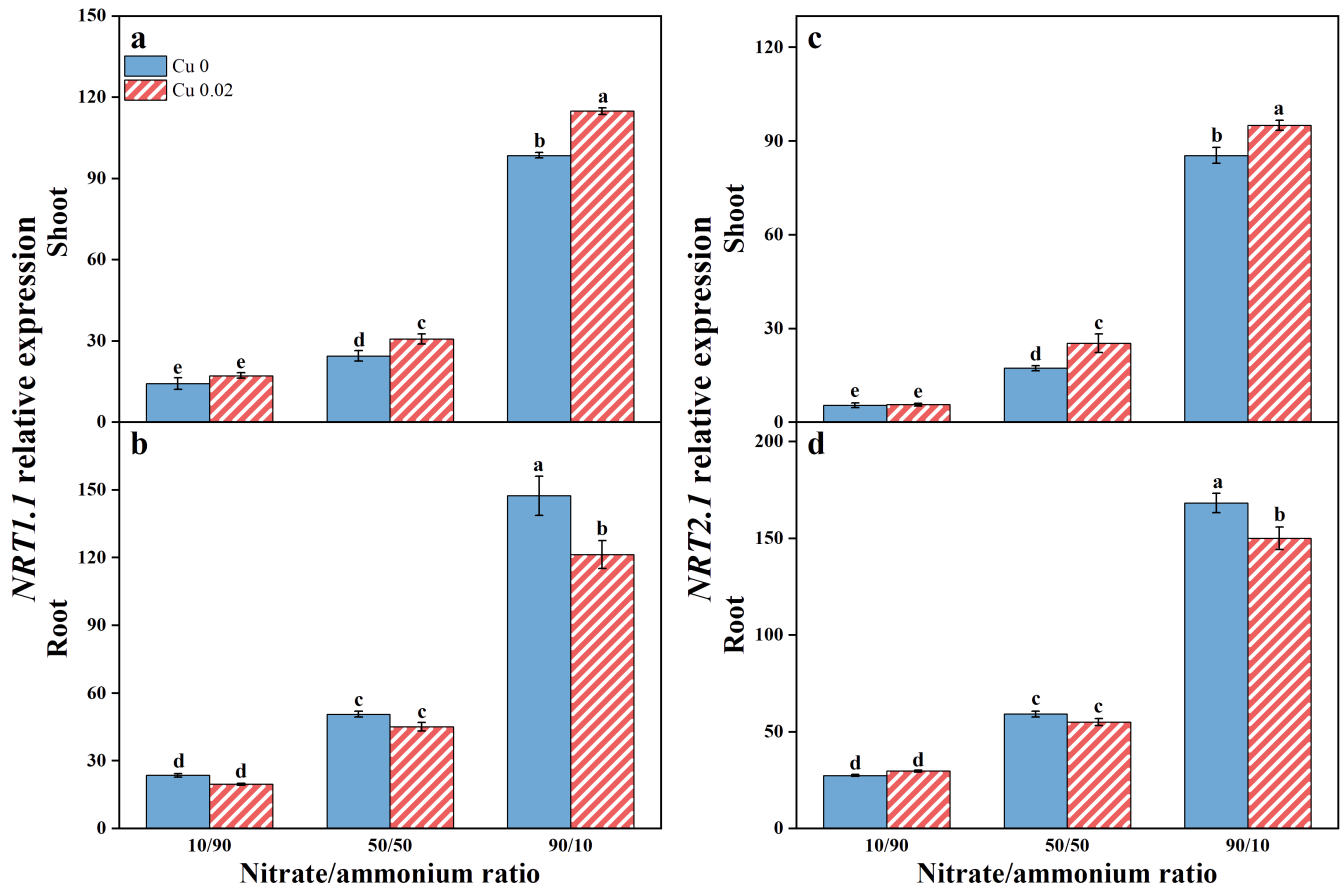
Effects of Cu on the expression of *NRT1.1* **(A, B)** and *NRT2.1* **(C, D)** in Chinese cabbage shoots and roots under three nitrate/ammonium ratios. Significance levels are denoted by distinct lowercase letters at *p* < 0.05.

Elevated nitrate proportions upregulated *NRT1.1* and *NRT2.1* expressions in both the shoot and root of Chinese cabbage (**Figure 2**). Notably, Cu application further enhanced *NRT1.1* and *NRT2.1* expressions in the shoots, particularly at the 50/50 and 90/10 nitrate/ammonium ratios (**Figures 2A, C**). Compared to the 10/90 ratio, *NRT1.1* expression increased by 25.32% and 16.57%, and *NRT2.1* expression increased by 46.69% and 11.28% at the 50/50 and 90/10 ratios, respectively. Conversely, Cu application downregulated *NRT1.1* and *NRT2.1* expressions in the roots (**Figures 2B, D**), especially at the 90/10 ratio, where reductions of 17.65% and 10.83%, respectively, were observed. To determine the primary expression site of *NRT1.1* and *NRT2.1*, the log of shoot-to-root transcription abundance was calculated following Tang et al. (2013). A log value < 0 indicates predominant root expression, while a positive value indicates shoot predominance. In this study, the log (shoot/root transcription abundance) for *NRT1.1* and *NRT2.1* was consistently < 0 across all treatments (**Supplementary Table S3**), indicating root-dominant expression unaffected by Cu application or nitrate/ammonium ratio.

### 3.3 NR activity and *NIA* gene expression

**Figure 3:**
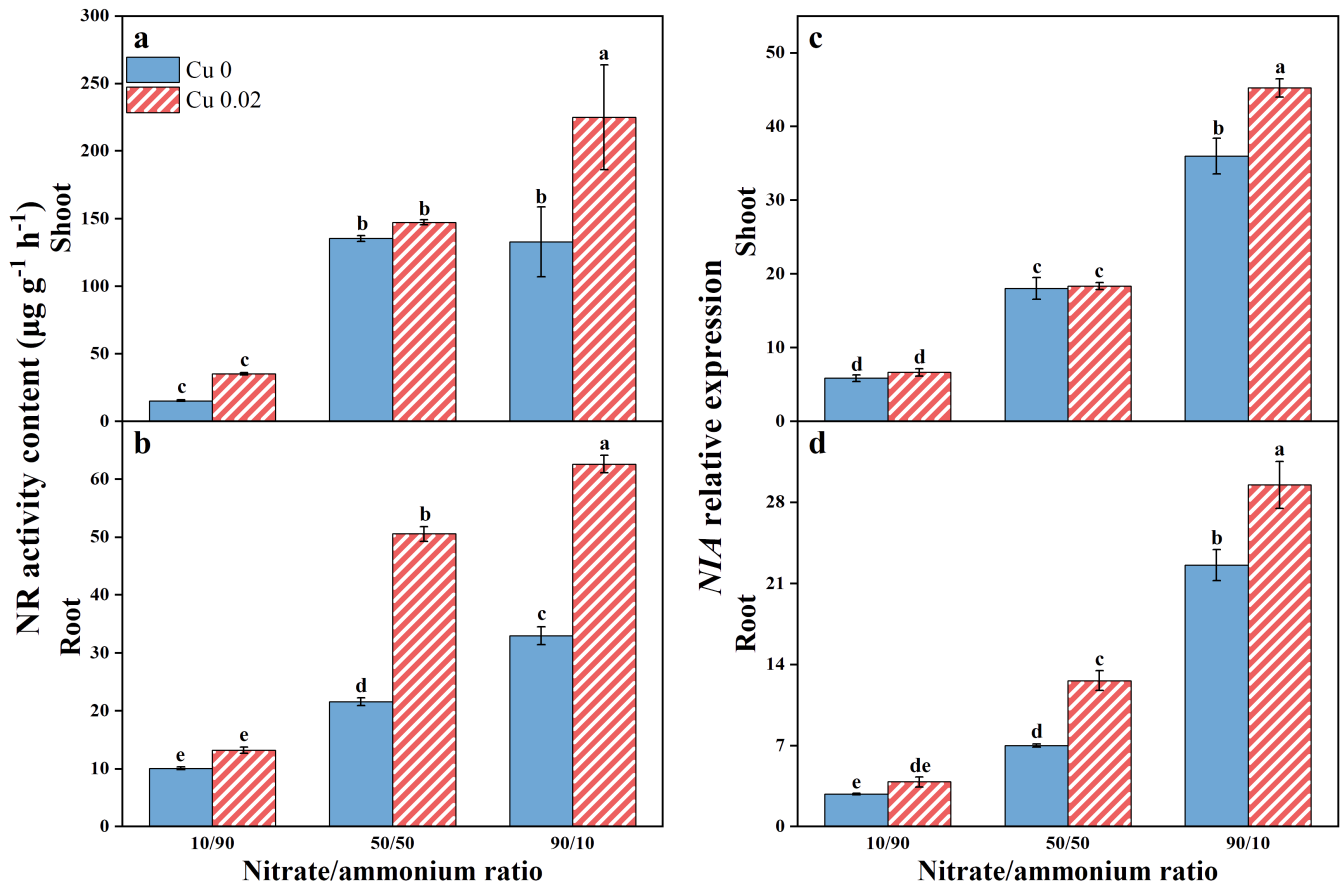
The effects of Cu on NR activity **(A, B)** and *NIA* gene expression **(C, D)** in Chinese cabbage shoots and roots under three nitrate/ammonium ratios. Significance levels are denoted by distinct lowercase letters at *p* < 0.05.

With increasing nitrate/ammonium ratios, NR activity in both shoots and roots showed an upward trend, more pronounced in the roots (**Figures 3A, B**). Cu application significantly increased NR activity in shoots by 69.41% at the 90/10 ratio and in roots by 134.64% and 89.85% at the 50/50 and 90/10 ratios, respectively, with statistically significant differences (*p* < 0.05).

The *NIA* gene expression pattern in Chinese cabbage mirrors the changes in NR enzyme activity, with relative expressions in shoots and roots increasing as the nitrate/ammonium ratios rise. Notably, Cu application significantly upregulated *NIA* expression in shoots by 25.71% at the 90/10 ratio (*p* < 0.05) and in roots by 80.19% and 30.65% at the 50/50 and 90/10 ratios, respectively (*p* < 0.05, **Figures 3C, D**). Furthermore, both NR activity and *NIA* expression were higher in shoots than in roots.

### 3.4 GS activity and its relative gene expression

**Figure 4:**
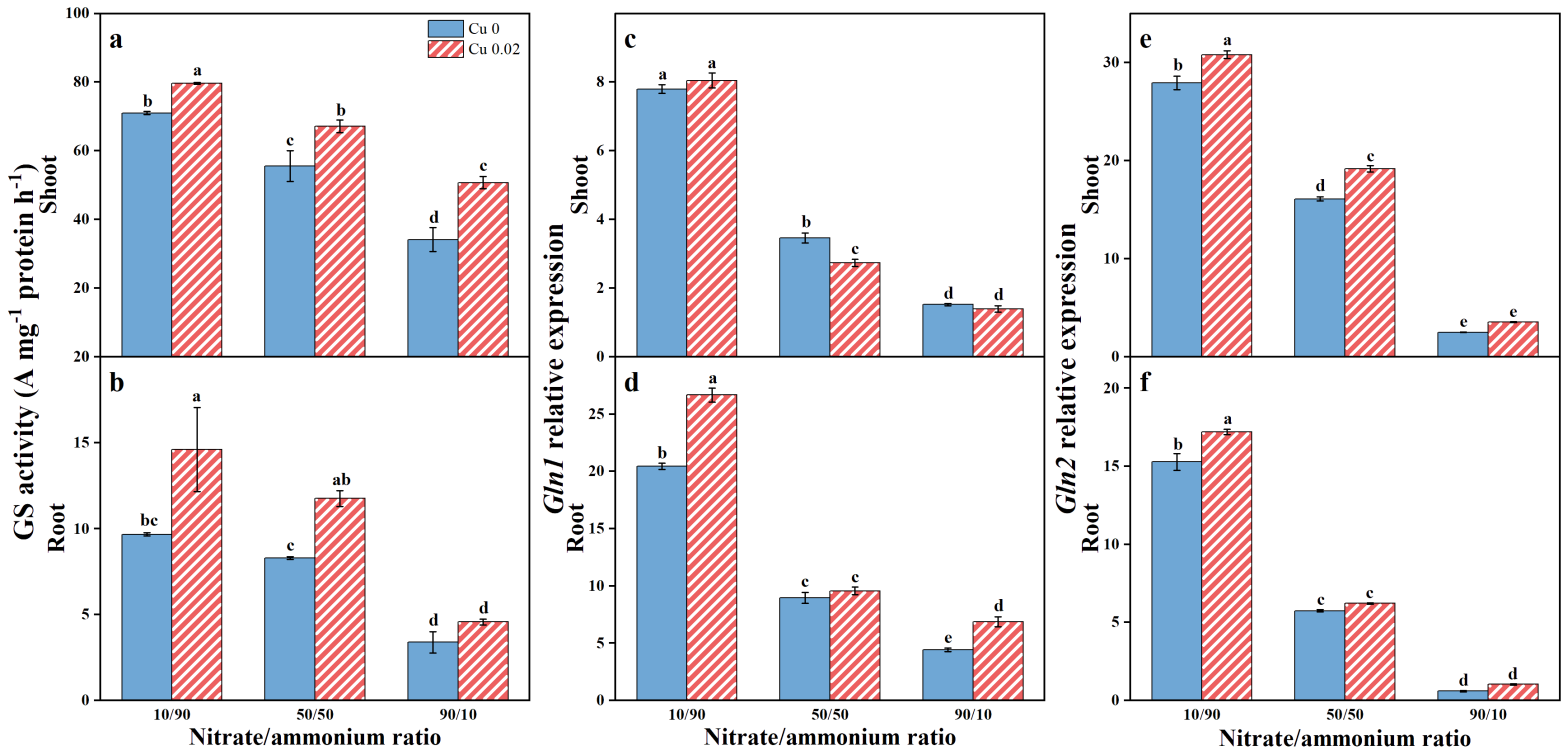
The effects of Cu on GS activity **(A, B)**, *Gln1* **(C, D)** and *Gln2* **(E, F)** expressions in Chinese cabbage shoots and roots under three nitrate/ammonium ratios. Significance levels are denoted by distinct lowercase letters at *p* < 0.05.

GS activity was assessed in Chinese cabbage tissues (**Figures 4A, B**), revealing generally higher enzyme activity in shoots compared to roots, regardless of Cu treatment or nitrate/ammonium ratio. Both Cu application and increased ammonium contribution significantly (*p* < 0.05) enhanced GS activity in shoots and roots, with the highest activity observed at Cu 0.02 under a nitrate/ammonium ratio of 10/90. Specifically, GS activity in shoots increased by 12.18%, 20.79%, and 48.65%, while in roots, it increased by 51.16%, 41.89%, and 35.04% under the 10/90, 50/50, and 90/10 ratios, respectively.

Similarly, *Gln1* and *Gln2* expression in Chinese cabbage responded sensitively to nitrate/ammonium ratios, with both genes showing upregulation as the ammonium proportion increased. As depicted in **Figure 4D**, Cu application significantly (*p* < 0.05) increased *Gln1* expression in roots by 30.41% and 55.01% under the 10/90 and 90/10 ratios, respectively. Additionally, *Gln2* expression in shoots significantly (*p* < 0.05) increased by 10.36% and 41.01% under the 10/90 and 50/50 ratios (**Figure 4E**), while in roots, it significantly (*p* < 0.05) increased by 12.63% under the 10/90 ratio (**Figure 4F**).

Regarding expression organs, regardless of the nitrate/ammonium ratio or the application of Cu, *Gln1* expression in shoots was lower than in roots. Conversely, *Gln2* expression showed the opposite trend, with higher expression in shoots than in roots, and these differences were statistically significant (*p* < 0.05).

### 3.5 Total free amino acid and soluble protein contents

**Figure 5:**
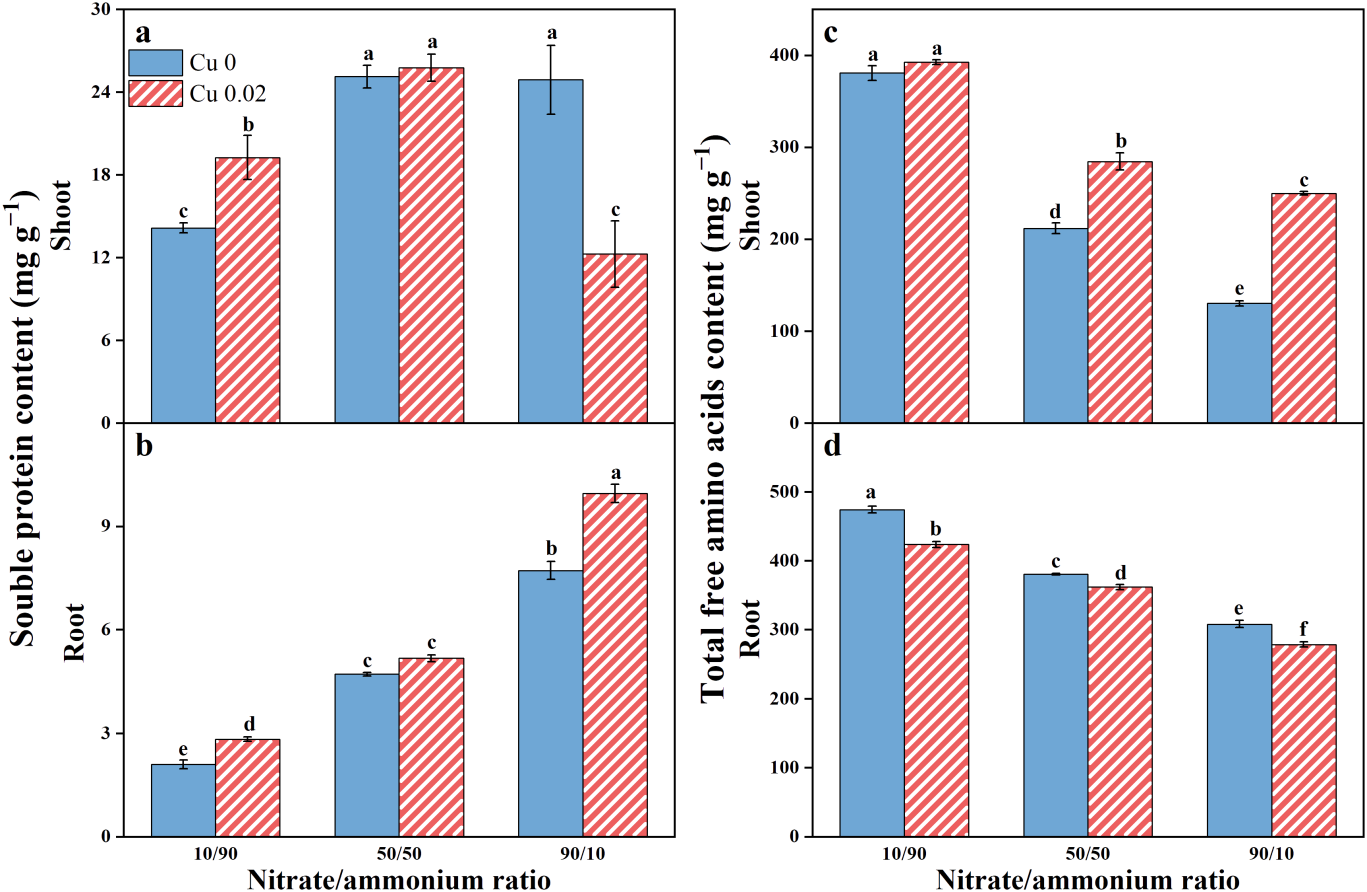
The effects of Cu on soluble protein **(A, B)** and total free amino acids **(C, D)** contents in Chinese cabbage shoots and roots under three nitrate/ammonium ratios. Significance levels are denoted by distinct lowercase letters at *p* < 0.05.

Cu application significantly increased soluble protein content in Chinese cabbage roots across all nitrate/ammonium ratios, showing a 34.89% and 28.94% increase (*p* < 0.05) under the 10/90 and 90/10 ratios, respectively (**Figure 5A**). However, the stimulatory effect of Cu on soluble protein content in shoots diminished with the rising nitrate proportion. It increased significantly (*p* < 0.05) by 36.05% under the 10/90 ratio, showed no significant difference under the 50/50 ratio, and decreased by 50.77% under the 90/10 ratio (*p* < 0.05, **Figure 5B**).

As the nitrate/ammonium ratio increased, total free amino acids content decreased in both shoots and roots of Chinese cabbage. However, Cu application increased total free amino acids content in shoots but decreased it in roots (**Figures 5C, D**).

### 3.6 The concentration and accumulation of N

**Figure 6:**
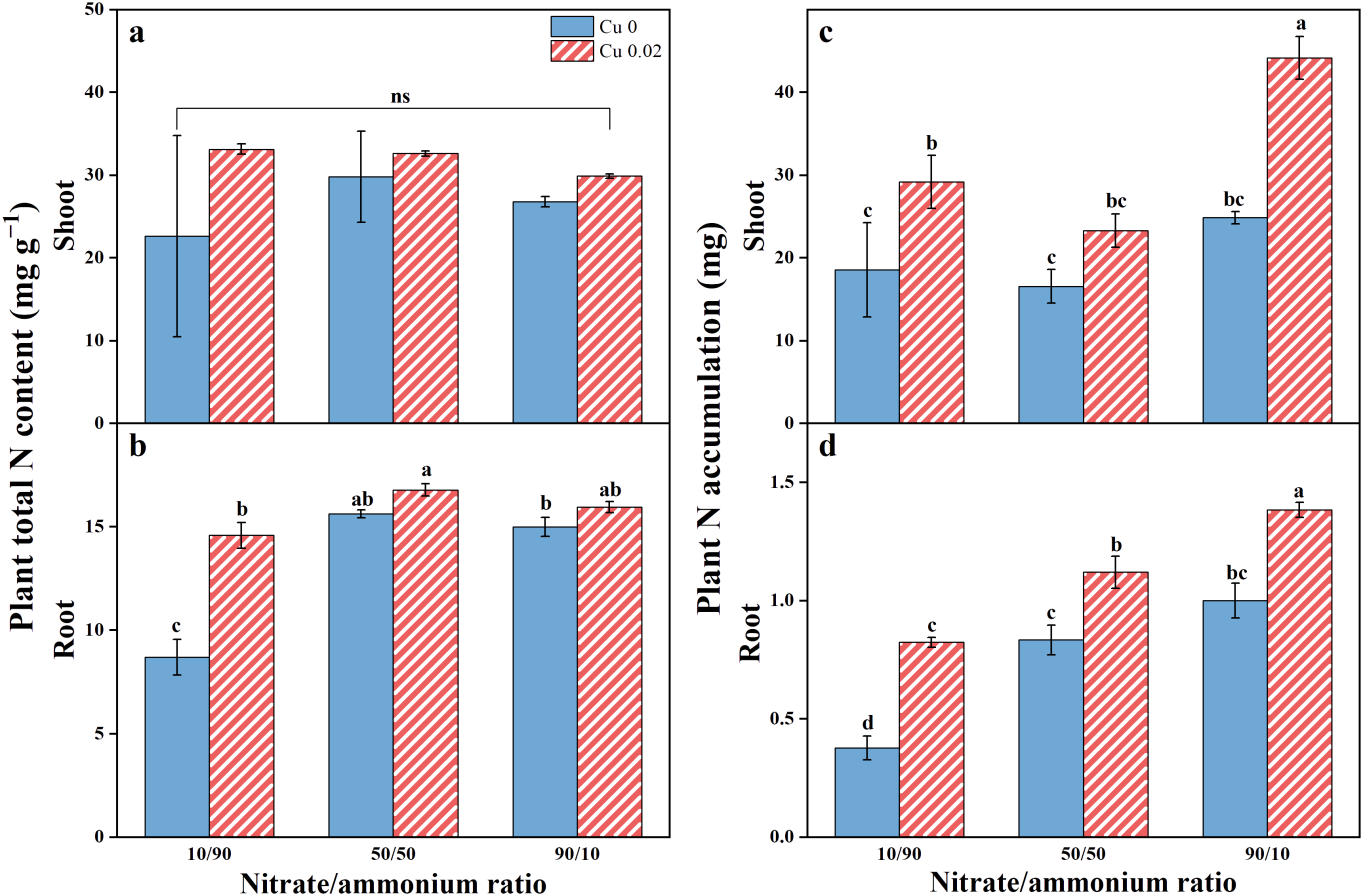
The effects of Cu on total N content **(A, B)** and N accumulation **(C, D)** in Chinese cabbage shoots and roots under three nitrate/ammonium ratios. Significance levels are denoted by distinct lowercase letters at *p* < 0.05. The ns represents insignificant change at the *p* < 0.05 level.

The total N concentration and accumulation in Chinese cabbage tissues are illustrated in **Figure 6**, showing higher levels in shoots compared to roots. Cu application enhanced these parameters in both shoots and roots, with a more notable impact on N accumulation. A shift in the nitrate/ammonium ratio from 10/90 to 90/10 increased N accumulation in shoots by 57.41% (*p* < 0.05), 40.79%, and 77.87% (*p* < 0.05), and roots by 118.58%, 34.40%, and 38.33% (*p* < 0.05). Notably, Cu application significantly increased the total N content in roots by 67.95% (*p* < 0.05) only under the 10/90 ratio.

### 3.7 Interaction effects of Cu and nitrate/ammonium ratio on tested parameters

**Table 4** reveals that the interactive effects of Cu application and nitrate/ammonium ratio significantly influence various parameters in both shoots and roots of Chinese cabbage. Specifically, in the shoots, Cu and nitrate/ammonium ratio significantly impacted nitrate content, ammonium content, total free amino acids, soluble protein, and the expression of *NIA*, *Gln1*, *Gln2*, *NRT1.1*, and *NRT2.1* genes. Conversely, in the roots, these interactive effects had a highly significant impact on total N content, nitrate content, ammonium content, total free amino acids, soluble protein, NR enzyme activity, and the expression of *NIA*, *Gln1*, *Gln2*, and *NRT2.1* genes, with a notable influence observed on *NRT1.1* expression.

**Table 4:** The *p*-values of the main effects and interaction effects of nitrate/ammonium ratio and Cu treatment.

Parameters	Shoot			Root		
	N ratios	Cu	Cu × N ratios	N ratios	Cu	Cu × N ratios
TN	0.855	0.331	0.796	<0.001	<0.001	<0.001
N accumulation	0.001	<0.001	0.159	<0.001	<0.001	0.371
Nitrate	<0.001	<0.001	<0.001	<0.001	<0.001	0.002
Ammonium	<0.001	0.021	<0.001	<0.001	<0.001	0.026
Total free amino acids	<0.001	<0.001	<0.001	<0.001	<0.001	0.007
Soluble protein	<0.001	0.106	<0.001	<0.001	<0.001	<0.001
NR	<0.001	0.021	0.109	<0.001	<0.001	<0.001
*NIA*	<0.001	0.007	0.008	<0.001	<0.001	0.042
GS	<0.001	<0.001	0.312	<0.001	0.003	0.241
*Gln1*	<0.001	<0.001	<0.001	<0.001	0.090	0.010
*Gln2*	<0.001	<0.001	0.009	<0.001	<0.001	0.030
*NRT1.1*	<0.001	<0.001	0.003	<0.001	0.007	0.053
*NRT2.1*	<0.001	0.002	0.047	<0.001	0.027	0.024

N ratios represent the main effect of nitrate/ammonium ratio, Cu represents the main effect of Cu application, and Cu × N ratios represent the interaction effect of both of these factors.

## 4 Discussion

The N metabolism in higher plants encompasses processes such as uptake, transport, assimilation, and remobilization (Liu et al., 2022). Our study reveals that Cu plays a direct or indirect role in these processes, particularly in the nitrate reduction process. This reduction, mediated by NR, is a crucial step in nitrate assimilation, influencing plant growth and organic nitrogen status (Xiong et al., 2006). NR acts as the initial enzyme in this process, reflecting the plant’s nitrate nutrition levels through its role in absorption, transport, and metabolism (Darnell and Hiss, 2006; Liu et al., 2017). The increase in NR activity and *NIA* expression in both shoots and roots following Cu addition (**Figure 3**) is likely due to Cu’s direct influence on NR through its essential role in Moco biosynthesis (Kaur et al., 2023). At the cellular level, Moco serves as a catalytic center for NR activation and nitrate reduction, with the final step of Moco biosynthesis associated with copper–dithiolate complex formation (Kuper et al., 2004). Additionally, NR activity is not only influenced by nitrate substrate (Chamizo-Ampudia et al., 2017) but also by factors like light intensity, plant hormones, and photoperiod (Sun et al., 2018). Carbohydrates, particularly sugars, affect NR expression (Xiong et al., 2006). Cu’s role in electron transfer during photosynthesis suggests it may indirectly enhance *NIA* expression and NR activity (**Figures 3C, D**) by influencing plant carbohydrate metabolism and photosynthesis (Kaur et al., 2023). These findings align with a recent study showing that Cu deficiency decreased N content and NR activity in rice (Du et al., 2022b). Given that our study has not yet examined the photosynthetic characteristics of Chinese cabbage, future research could focus on the influence of Cu on plant photosynthetic performance and carbohydrate production under varying nitrate/ammonium ratios. This would provide further evidence to support the hypothesis.

Under Cu addition, nitrate content and the expressions of *NRT1.1* and *NRT 2.1* decreased in Chinese cabbage roots, but increased in the shoots (**Figures 1**, **2**), indicating Cu may enhance nitrate transport. Most nitrate absorbed by plants is transported to aerial parts, while a small portion is stored in root vacuoles for osmotic regulation (Kozlowski et al., 2016). Leafy vegetables predominantly accumulate nitrate in their leaves (Colla et al., 2018). Thus, nitrate content in Chinese cabbage shoots was significantly higher than in roots (**Figures 1A, B**). Nitrate absorption and transportation are primarily mediated by membrane transporters, such as *NRT1* and *NRT2* families. Our study hypothesizes that Cu increases NR activity in Chinese cabbage roots (**Figure 3B**), accelerating nitrate reduction, which significantly decreases nitrate content (**Figure 1B**). This reduction provides signals to downregulate the expression of *NRT1.1* and *NRT2.1* (**Figures 2B, D**), consistent with previous findings (Krapp et al., 2014; Chamizo-Ampudia et al., 2017). Additionally, optimizing Cu supply enhances root growth and development, enhancing nutrient absorption and transport capacity of roots (Ameh and Sayes, 2019). The enhanced root biomass of Chinese cabbage with Cu application (**Table 3**) supports the proposed hypothesis. Regardless of Cu application, NR activity, *NIA* expression, and nitrate content in Chinese cabbage shoots were significantly higher than in roots (**Figures 1A, B**, **3**), suggesting that nitrate reduction by NR predominantly occurs in the shoots. This is because most nitrate absorbed by roots is transported to the shoots for reduction in mesophyll cells (MCs), although some can be reduced to ammonium in roots (Liu et al., 2022). However, *NRT1.1* and *NRT2.1* transporters, predominantly expressed in Chinese cabbage roots, align with findings of their positive correlation with nitrate uptake in plant roots (Tang et al., 2013; Chamizo-Ampudia et al., 2017). Chinese cabbage’s nitrate preference enhances root nitrate absorption capability (Zhang et al., 2023). In *Arabidopsis*, *AtNRT1.1* and *AtNRT2.1* mediate low-affinity and high-affinity nitrate transport systems (Tsay et al., 2007; Miller et al., 2009). This study used nutrient solutions with nitrate concentrations of 1.5, 7.5, and 13.5 mM. *NRT1.1* and *NRT2.1* expressions significantly increased with higher nitrate concentrations, with no significant difference in expression levels, except for a slight increase in *NRT1.1* expression over *NRT2.1* in shoots, indicating both are inducible by higher nitrate concentrations (Okamoto et al., 2003).

In plants, ammonium, sourced from nitrate reduction and root absorption, is the primary source for N assimilation, predominantly transported to aerial parts for growth and development (Wei et al., 2020). This assimilation is catalyzed by the GS-GOGAT cycle, with GS acting as a crucial enzyme and ammonium reservoir (Hachiya et al., 2021). We found that Cu application increased GS activity in Chinese cabbage shoots and roots (**Figure 4**), aligning with previous studies on other crops (Llorens et al., 2000; Zhang et al., 2014; de Souza Junior et al., 2018). The upregulation of *Gln1* and *Gln2* expressions explained this enhancement (**Figure 4**). These isoforms are distributed across various plant organs and perform diverse functions (Martin et al., 2006). *Gln1* is primarily located in roots and older leaves, playing a role in ammonium assimilation in shoots and recycling ammonium released during protein degradation in leaf senescence (Ingargiola et al., 2023). Ammonium significantly increased the accumulation of *ZmGLN1.1* and *ZmGLN1.5* in maize roots and the expression of *TaGS1.1* and *TaGS1.3* in wheat roots (Prinsi and Espen, 2015; Wei et al., 2020; Liu et al., 2022). However, *Gln2* primarily absorbs ammonium derived from nitrate reduction in chloroplasts or ammonia released during photorespiration (Perez-Delgado et al., 2016). *Arabidopsis AtGLN2* is present in both mitochondria and chloroplasts of MCs, with its GS activity in these organelles enhanced by photorespiratory stress (Liu et al., 2022). *Gln1* and *Gln2* are predominantly expressed in the roots and shoots of Chinese cabbage, respectively (**Figure 4**), consistent with previous findings. Notably, *Gln2*’s response to Cu is more consistent, as Cu upregulates *Gln2* expression in both roots and shoots across all nitrate/ammonium ratios (**Figures 4E, F**). This could be due to Cu’s role in enhancing NR activity and facilitating nitrate reduction, potentially leading to *Gln2* upregulation. Another hypothesis is that Cu-induced upregulation of *Gln2* may be related to its role in assimilating ammonia released during photorespiration (Perez-Delgado et al., 2016), potentially enhancing photosynthesis (Zunaira et al., 2020). This hypothesis warrants further investigation. In contrast, *Gln1*, encoded by a multigene family, plays a more complex role in various biological processes such as primary N assimilation, remobilization during leaf senescence, and grain filling (Thomsen et al., 2014; Liu et al., 2022). This complexity may explain the weaker effect of Cu on *Gln1* expression.

Ammonium is transformed into proteins, amino acids, chlorophyll, and other nitrogen-containing organic compounds (Perez-Delgado et al., 2016). Cu is crucial in promoting plant growth and development by participating in protein transport, regulating photosynthetic electron transport, and hormone signal transduction (Ameh and Sayes, 2019). These functions collectively enhance plant N assimilation and conversion to organic N compounds. Cu in plant tissues forms complexes with amino acids in the xylem before being transported to the aerial parts, thereby increasing the total free amino acid content in plant tissues (Liao et al., 2000; Mazen, 2004; Xiong et al., 2006), thus promoting protein synthesis (Rehman et al., 2019). In our study, Cu application facilitated the transfer of total free amino acids from roots to shoots, increasing the soluble protein content in roots (**Figure 5**). This supports the indirect role of Cu in the conversion of ammonium to organic N compounds in Chinese cabbage. Furthermore, excess external ammonium can disrupt the tricarboxylic acid (TCA) cycle, leading to ammonium accumulation in plant tissues (Lasa et al., 2002). Our findings confirmed that the 10/90 nitrate/ammonium ratio indeed resulted in ammonium accumulation in the major metabolic organs of Chinese cabbage. However, Cu application significantly decreased ammonium levels in both roots and shoots of Chinese cabbage, increasing soluble protein content and enhancing GS activity. Consequently, this upregulated the expression of *Gln1* and *Gln2* genes, thereby significantly increasing N accumulation in both shoots and roots. This process promoted ammonium assimilation and conversion to organic N, alleviating the phytotoxic effects of high ammonium. However, under a 90/10 nitrate/ammonium ratio, Cu application caused a significant decrease in shoot soluble protein content (**Figure 5A**). We hypothesize that this was due to the increased biomass and nitrate content in Chinese cabbage shoots under Cu application in a high-nitrate environment (**Table 2**, **Figure 1A**). This may have expedited leaf senescence, reducing primary N assimilation capacity and increasing protein and nucleic acid degradation (Have et al., 2017; Liu et al., 2022). Chinese cabbage, a nitrate-loving crop, may exhibit premature senescence and nitrate accumulation in edible parts under high nitrate conditions (Zhang et al., 2023). Therefore, excessive nitrate is not always beneficial for its growth.

Additionally, the impact of Cu on N uptake and assimilation in Chinese cabbage is significantly affected by the nitrate/ammonium ratio, a finding that has been documented in various plant species, including lettuce (Du et al., 2022a), Chinese cabbage (Xiong et al., 2006), tomato (Liu et al., 2017), and Tanzania guinea grass (de Souza Junior et al., 2018). However, these studies have primarily focused on the environmental effects of nitrate/ammonium ratios in mitigating Cu pollution. In contrast, our research highlights the regulatory role of optimal Cu concentrations on N nutrition in Chinese cabbage. Our study revealed significant interactive effects of Cu and nitrate/ammonium ratios on multiple parameters (**Table 4**), supported by gene expression levels. Under nitrate conditions, the impact of Cu on N metabolism is more pronounced, aligning with previous studies (Llorens et al., 2000). This finding enhances our understanding of the crucial role of Cu (at appropriate concentration) in N metabolism, a topic less frequently explored in the literature. Future studies will aim to elucidate the regulatory mechanisms of Cu on plant N uptake and utilization using proteomics and metabolomics techniques, with the goal of identifying potential relationships between metabolites and metabolic pathways.

## 5 Conclusion

Our comparative analyses demonstrated that Cu affects both growth and N metabolism in Chinese cabbage under varying nitrate/ammonium ratios. The 0.02 mg L^−1^ Cu application enhances NR activity, which promotes nitrate reduction in roots and its subsequent transport to shoots, resulting in nitrate accumulation in shoots. Additionally, Cu increases GS activity, thereby supporting the GS-GOGAT cycle and primary N assimilation. This enhancement facilitates the transfer of free amino acids from shoots to roots, resulting in greater soluble protein accumulation in roots, ultimately improving N accumulation and biomass production. Furthermore, key genes involved in plant N metabolism, such as *NRT1.1*, *NRT2.1*, *NIA*, *Gln1*, and *Gln2*, respond variably to Cu application. Additionally, the effect of Cu on N metabolism is further modulated by different nitrate/ammonium ratios in the nutrient solution. These findings highlight Cu’s essential role in N uptake, transport, and assimilation in higher plants, elucidating the complex interplay between Cu and nitrate/ammonium ratios, and providing insights into optimal ratios for the safe cultivation of Chinese cabbage.

## Data Availability

The original contributions presented in the study are included in the article/**Supplementary Material**. Further inquiries can be directed to the corresponding author.
