# Supplementary material for: Effect of copper on nitrogen uptake, transportation, assimilation processes, and related gene expression in Chinese cabbage [Brassica campestris L. ssp. Chinensis (L.)] under various nitrate-to-ammonium ratios
Source: Front Plant Sci. 2024 Sep 25;15:1427720. doi: 10.3389/fpls.2024.1427720 (PMC11461266; doi:10.3389/fpls.2024.1427720)
Supplement: Supplementary file 1 [file DataSheet1.pdf]

Supplementary Materials

Effect of copper on nitrogen uptake, transportation, assimilation processes, and related gene expression in Chinese cabbage [*Brassica campestris* L. ssp. *Chinensis* (L.)] under various nitrate-to-ammonium ratios

Xin Wen [1,2,3]*, Peiran Xu [3†], Yafang Tang [3†], Hang Zhong [3], Pan Chen [3], Zhenhao Zhu [3], Xinya Zhang [3], Xiaohui Zhang [3], Aoran Du [4]

[1]Zhejiang Ecological Civilization Academy, Anji, Zhejiang, China

[2]College of Environmental and Resource Sciences, Zhejiang University, Hangzhou, Zhejiang, China

[3]Hubei Key Laboratory of Quality Control of Characteristic Fruits and Vegetables, Hubei Engineering University, Xiaogan, Hubei, China

[4]School of Management, Minzu University of China, Beijing, China

*** Correspondence:**

Corresponding Author

Dr. Xin Wen, wx0630@zju.edu.cn

[†] These authors contributed equally to this work and share first authorship.

Number of tables: 4

Number of figures: 7

Number of pages: 8

Contents

Tables:

Table S1 The reaction mixture used in qRT-PCR

Table S2 The amplification efficiency of the primers for genes

Table S3 The logarithm of shoot-to-root transcription abundance of *NRT1.1* and *NRT2.1* under different treatments

Table S4 The timing of the various cultivation measures for Chinese cabbage during the course of the experiment

Figures:

Figure S1 The dissociation curve of the primer for *NRT1.1*

Figure S2 The dissociation curve of the primer for *NRT2.1*

Figure S3 The dissociation curve of the primer for *NIA*

Figure S4 The dissociation curve of the primer for *Gln1*

Figure S5 The dissociation curve of the primer for *Gln2*

Figure S6 The dissociation curve of the primer for *Actin*

Figure S7 Effects of Cu on the growth of Chinese cabbage under different nitrate/ammonium ratios

Table S1 The reaction mixture used in qRT-PCR

**The reaction mixture**		**Thermal profile**
SYBR Green	10 μL	
Forward Primer	0.8 μL	95 °C for 30 s;
Reverse Primer	0.8 μL	44 cycles × (95℃, 10 s; 55℃, 20 s; 72℃, 20 s);
synthesized cDNA	2 μL	95 °C for 10 s.
dd H_2O	Up to 20 μL	

Note: The kit used was SYBR Green PCR Master Mix Kit (TOYOBO, Japan)

Table S2 The amplification efficiency of the primers for genes

Gene	Slope	Y-Inter	R^2	Efficiency %
NRT1.1	-3.38	10.43	0.999	97.58
NRT2.1	-3.39	9.41	0.9985	96.08
NIA	-3.41	6.67	0.9997	93.33
Gln1	-3.31	8.78	0.9988	98.43
Gln2	-3.42	8.22	0.9974	96.04
Actin	-3.46	8.77	0.999	100.13

Table S3 The logarithm of shoot-to-root transcription abundance of *NRT1.1* and *NRT2.1* under different treatments

Transporters	Nitrate/ammonium ratios	Cu levels	shoot/root	log (shoot/root)
NRT1.1	10/90	Cu 0	0.60	−0.22
	10/90	Cu 0.02	0.88	−0.06
	50/50	Cu 0	0.48	−0.31
	50/50	Cu 0.02	0.68	−0.17
	90/10	Cu 0	0.67	−0.17
	90/10	Cu 0.02	0.95	−0.02
NRT2.1	10/90	Cu 0	0.20	−0.70
	10/90	Cu 0.02	0.19	−0.72
	50/50	Cu 0	0.29	−0.54
	50/50	Cu 0.02	0.46	−0.34
	90/10	Cu 0	0.51	−0.29
	90/10	Cu 0.02	0.63	−0.20

Note: shoot/root represent shoot-to-root transcription abundance of the gene, log (shoot/root) represent logarithm of shoot-to-root transcription abundance.

Table S4 The timing of the various cultivation measures for Chinese cabbage during the course of the experiment.

Cultivation process	Date
Seed germination	October 19, 2021
Seedlings transfer to the half-strength nutrient solution	October 24, 2021
Seedlings transfer to the experimental nutrient solution	November 7, 2021
Sampling collected	December 22, 2021

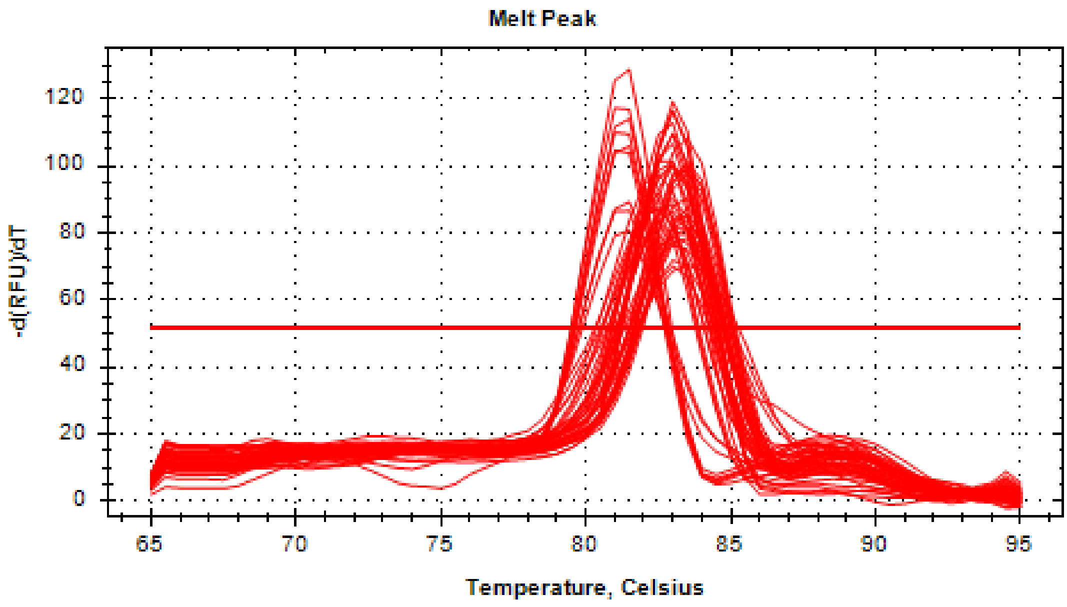

Figure S1 The dissociation curve of the primer for *NRT1.1*

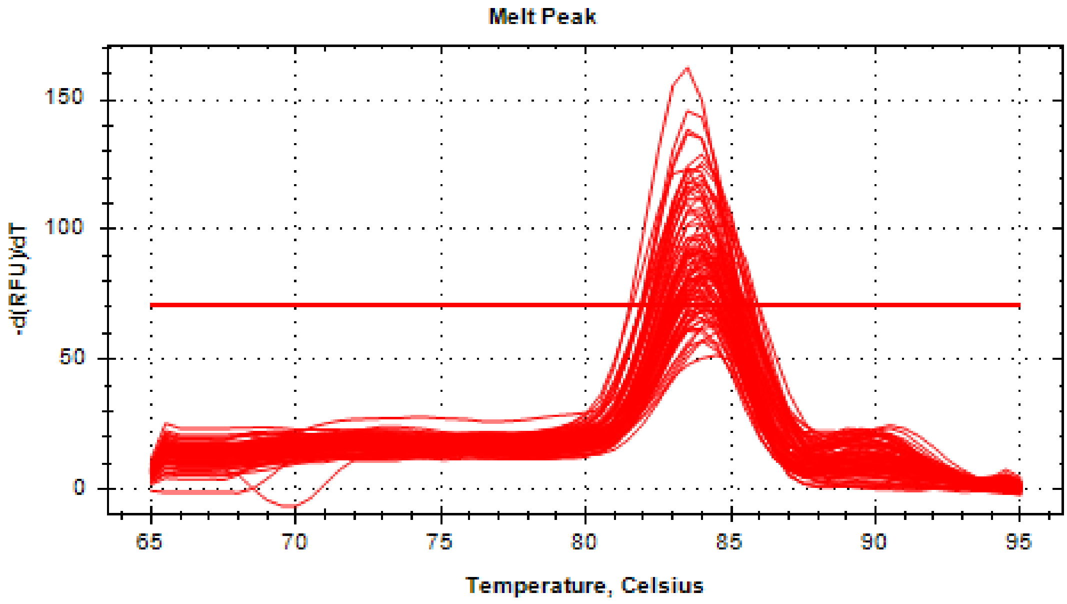

Figure S2 The dissociation curve of the primer for *NRT2.1*

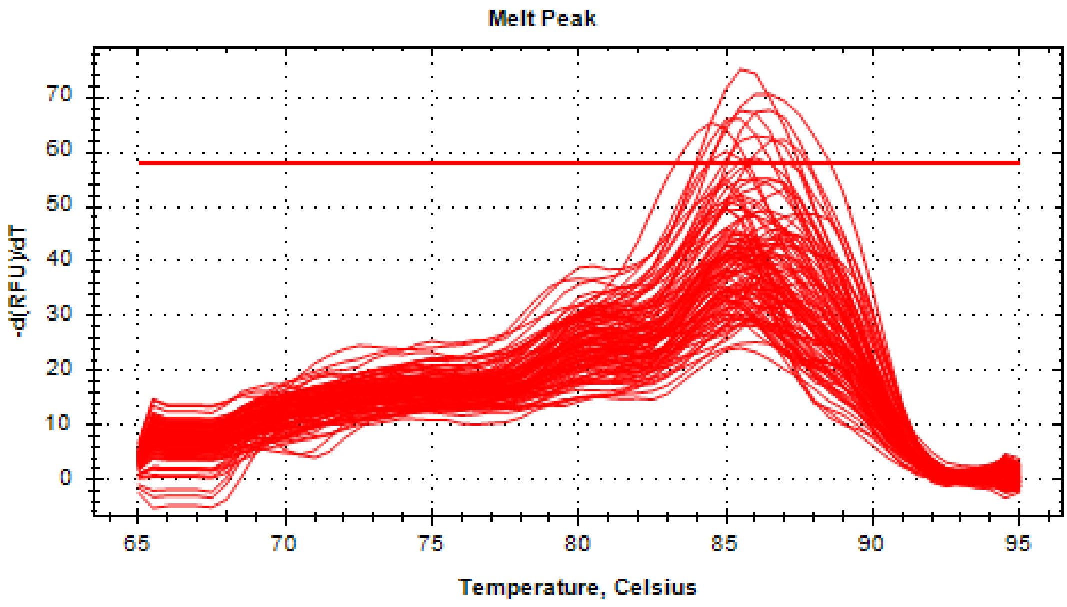

Figure S3 The dissociation curve of the primer for *NIA*

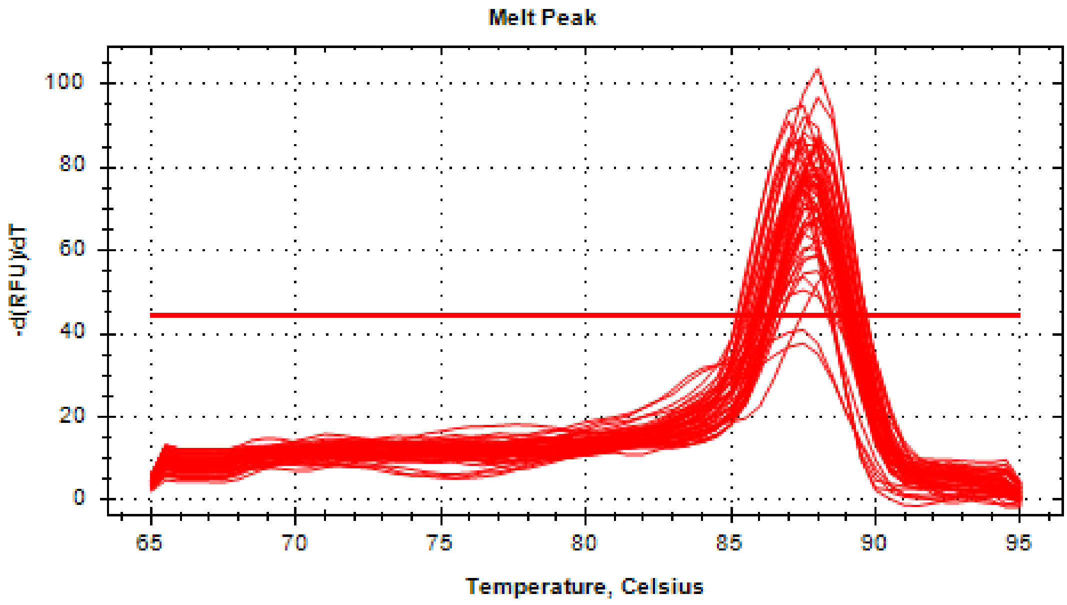

Figure S4 The dissociation curve of the primer for *Gln1*

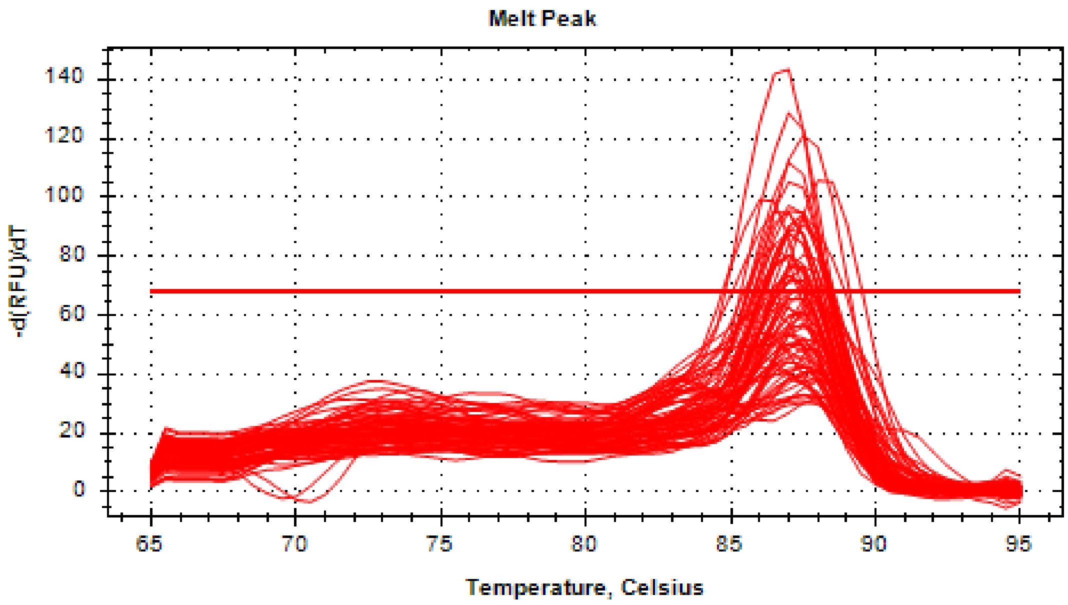

Figure S5 The dissociation curve of the primer for *Gln2*

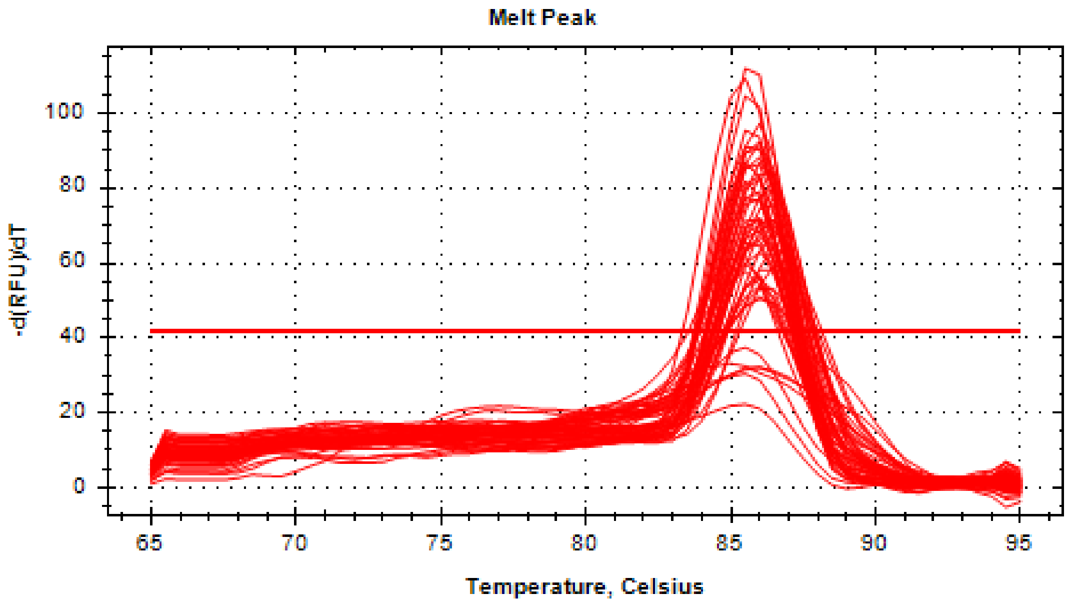

Figure S6 The dissociation curve of the primer for *Actin*

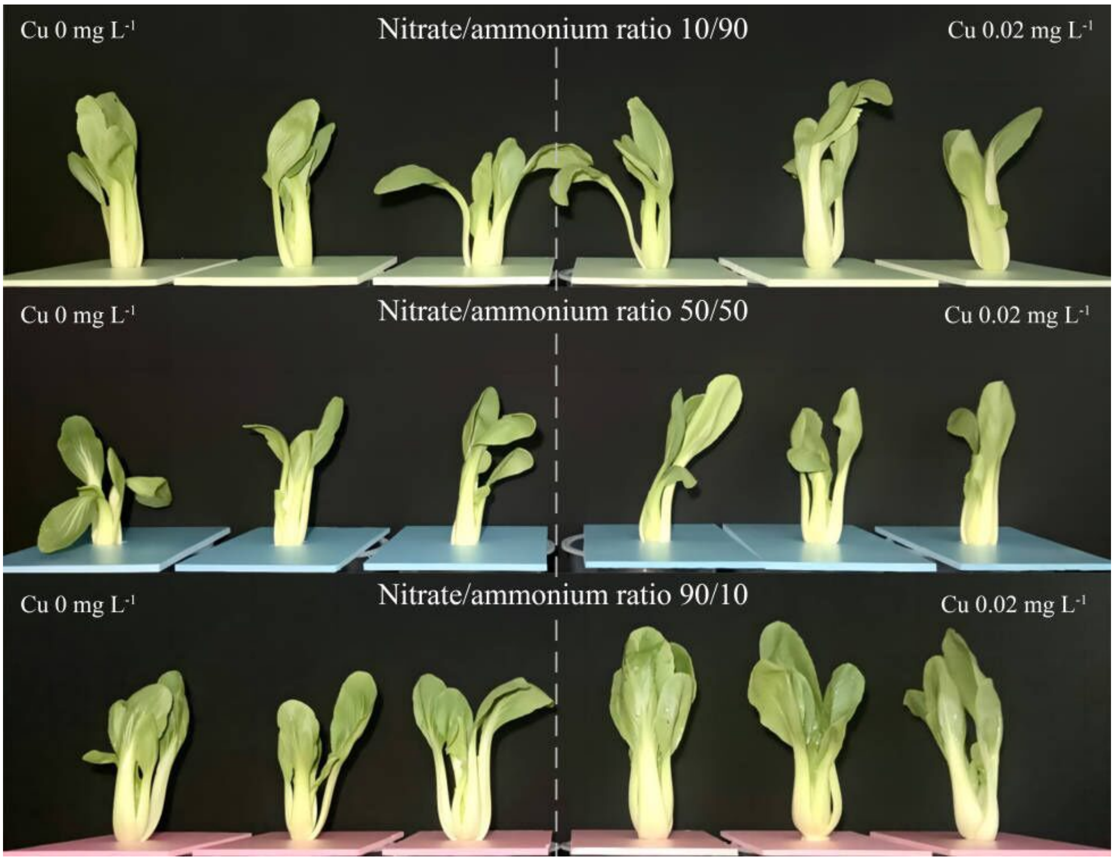

Figure S7 Effects of Cu on the growth of Chinese cabbage under different nitrate/ammonium ratios
